# Cold atmospheric plasma causes a calcium influx in melanoma cells triggering CAP-induced senescence

**DOI:** 10.1038/s41598-018-28443-5

**Published:** 2018-07-03

**Authors:** Christin Schneider, Lisa Gebhardt, Stephanie Arndt, Sigrid Karrer, Julia L. Zimmermann, Michael J. M. Fischer, Anja-Katrin Bosserhoff

**Affiliations:** 1 Institute of Biochemistry, Emil-Fischer-Center, University of Erlangen-Nürnberg, Erlangen, Germany; 2 Institute of Physiology and Pathophysiology, University of Erlangen-Nürnberg, Erlangen, Germany; 3 Department of Dermatology, University Hospital of Regensburg, Regensburg, Germany; 4 Terraplasma GmbH, Garching, Germany; 5 Institute of Physiology, Medical University of Vienna, Vienna, Austria; 6 Comprehensive Cancer Center (CCC) Erlangen-EMN, Erlangen, Germany

## Abstract

Cold atmospheric plasma (CAP) is a promising approach in anti-cancer therapy, eliminating cancer cells with high selectivity. However, the molecular mechanisms of CAP action are poorly understood. In this study, we investigated CAP effects on calcium homeostasis in melanoma cells. We observed increased cytoplasmic calcium after CAP treatment, which also occurred in the absence of extracellular calcium, indicating the majority of the calcium increase originates from intracellular stores. Application of previously CAP-exposed extracellular solutions also induced cytoplasmic calcium elevations. A substantial fraction of this effect remained when the application was delayed for one hour, indicating the chemical stability of the activating agent(s). Addition of ryanodine and cyclosporin A indicate the involvement of the endoplasmatic reticulum and the mitochondria. Inhibition of the cytoplasmic calcium elevation by the intracellular chelator BAPTA blocked CAP-induced senescence. This finding helps to understand the molecular influence and the mode of action of CAP on tumor cells.

## Introduction

Plasma is ionized gas, which is composed of reactive oxygen (ROS) and nitrogen (RNS) species, charged particles and an optical emission also in the UV range. Plasma commonly has high temperatures, however it has become possible to produce so called cold atmospheric plasma (CAP) which has almost room temperature. Since then, the use of CAP in medical applications has gained importance^1,2^.

There are three major types of CAP: indirect plasma, direct plasma and “hybrid” plasma^3^. For the generation of direct plasma, the sample itself serves as an electrode and is directly involved in CAP generation^4,5^. Often used is the so-called dielectric barrier discharge (DBD) device with electrodes separated by an insulating barrier^4^. Indirect plasma is produced between two electrodes and transported to the sample through a gas flow, commonly consisting of an inert gas like helium or argon. The absence of a barrier between the electrodes results in stronger discharges as well as UV radiation compared to direct plasma^4^. “Hybrid” plasma is produced directly, but in contrast to DBD, the sample does not serve as a counter electrode^6^. A grounded mesh electrode prohibits any current flow through the sample^7^. For this study we used “hybrid” plasma produced by a portable plasma device (miniFlatPlaSter) based on the Surface Micro Discharge (SMD) technology. The plasma is generated with the surrounding air through a range of microdischarges^7,8^. The use of diverse plasma sources results in differences regarding the plasma components, which still makes comparisons of the various plasma effects on different organisms and cell types difficult. Further, the development of new plasma sources and the efforts to optimize devices for specific applications are ongoing.

In the last few years, CAP has also been proposed as a potential new anti-cancer therapy. Investigations with different cancer cell lines and devices showed that CAP induces apoptosis^9–11^ and suppresses cell migration and invasion^12,13^. In some studies induction of necrosis has also been observed^14,15^. It is of particular interest that in contrast to conventional anti-cancer therapies CAP exposure which affected cancer cells with high selectivity was reported^11,16,17^. We observed dose-dependent effects on malignant melanoma cells by CAP generated with the SMD technique^18^. CAP treatment for 2 min induced DNA damage and resulted in apoptosis of the melanoma cells. Interestingly, CAP caused apoptosis in only 9% of normal human epidermal melanocytes (NHEMs)^18^. Furthermore, a shorter CAP exposure of 1 min led to induction of senescence without detection of DNA damage and apoptosis. Dose-dependent effects of CAP produced by a SMD device could also be observed on squamous head and neck cancer cells, leading to reduction of cell viability and DNA damage^19^.

Several studies described CAP-induced anti-cancer effects to be caused by ROS and RNS^20,21^. For ROS, it is well known that there is a close relation to calcium (Ca^2+^) signaling^22^. Ca^2+^ is an important second messenger regulating various cellular processes that are involved in tumorigenesis and tumor progression, such as angiogenesis^23^, tumor invasion^24^ and tumor growth^23^. Ca^2+^ is also implicated in apoptosis by triggering cytochrome c release from mitochondria^25,26^. A relatively new field is the involvement of Ca^2+^ in cellular senescence, for instance Ca^2+^ homeostasis can regulate telomerase activity^27–29^. Since CAP induces apoptosis and senescence^18^ the aim of this study was to investigate the impact of CAP on transient cytoplasmic Ca^2+^ elevation and downstream events in malignant melanoma cells.

## Results

### CAP causes a dose-dependent and delayed Ca^2+^ influx

**Figure 1:**
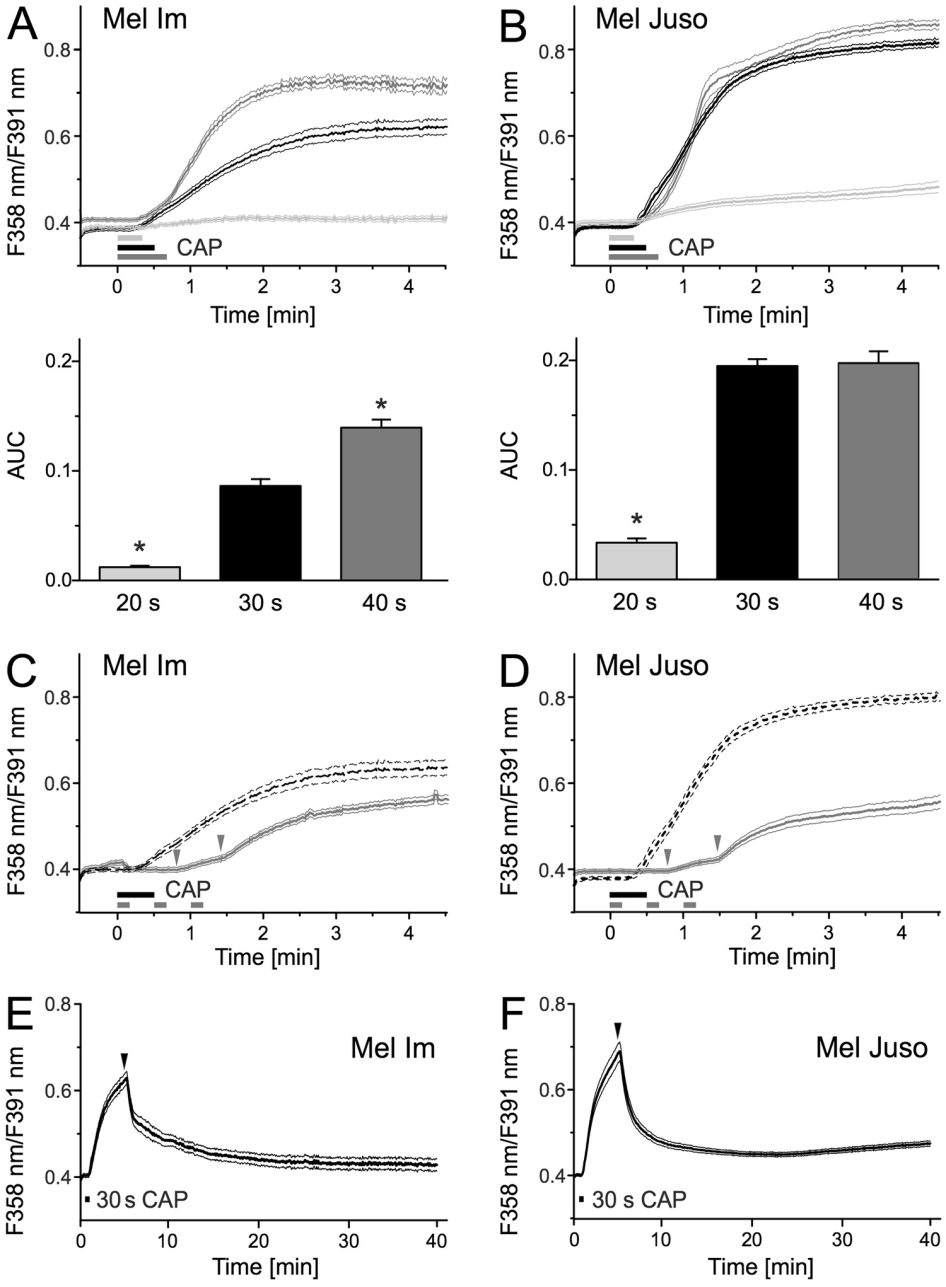
CAP treatment leads to a delayed Ca2^+^ influx. Cytoplasmic Ca^2+^ time courses were measured using fura-2 AM. CAP exposure for 20 s, 30 s and 40 s elevated cytoplasmic Ca^2+^ in Mel Im (**A**, n = 500–660) and Mel Juso (**B**, n** = **280–503). The area under the curve (AUC) of the first 90 s of this response was quantified in the bar charts. (**C**) Mel Im (n = 299) and (**D**) Mel Juso (n = 261) show the response pattern more clearly when the 30 s exposure (as in **A**,**B** for reference, dotted line) is fractionated into 3 times with 10 s CAP as indicated by the grey bars. The grey arrows mark the delay of the Ca^2+^ response. (**E**,**F**) Long-term measurement of cytoplasmic Ca^2+^ after treatment of Mel Im ((**E**), n = 275) and Mel Juso ((**F**), n = 353) with 30 s CAP. PbECS (1 ml) was added 5 minutes after the start of recording, represented as black arrows. Data are shown as mean and 99% confidence interval. N indicates the investigated number of cells.

We investigated the effect of CAP on changes in the cytoplasmic Ca^2+^ concentration in the melanoma cell lines Mel Im (derived from a metastasis) and Mel Juso (derived from a primary tumor) by using the fluorescence dye fura-2 AM. We first treated the cells during Ca^2+^ imaging directly with different CAP doses (20 s, 30 s, and 40 s). CAP exposure for 20 s and longer caused an increase in intracellular Ca^2+^ (p < 0.001 each, n = 500–660 cells for Mel Im, n = 280–503 cells for Mel Juso, t-test, single sample vs. no change, Fig. 1A,B). The Ca^2+^ response in both cell lines was positively associated with the duration of the treatment period to CAP (R = 0.34, p < 0.001, Mel Im, R = 0.56, p < 0.001, Mel Juso, product-momentum correlation, Fig. 1A,B). The Ca^2+^ response of Mel Im cells gradually increased with increasing CAP dose, whereas Mel Juso cells already showed a maximum intracellular Ca^2+^ elevation after 30 s CAP treatment. Treatment with longer exposure times is limited by cells detaching from the culture dish upon longer exposures.

Continuous CAP treatment elevated intracellular Ca^2+^ after 18 s and 15 s, respectively (Mel Juso and Mel Im, compared to the 95% confidence interval of the last 30 s). Interestingly, threefold CAP treatment of 10 s had an additive effect. There was no detectable change in cytoplasmic Ca^2+^ after the first 10 s CAP treatment but both further 10 s CAP exposures caused a Ca^2+^ response with a delay of 31 s and 30 s after starting the fractionated CAP treatment of Mel Im and Mel Juso, respectively (Fig. 1C,D). In the described experiments the cytoplasmic Ca^2+^ elevation occurred for a prolonged period, further developing or at least not recovering to a relevant degree within 5 minutes, as it is common for other means of stimulation. In measurements with a longer follow up after 30 s CAP, addition of phosphate buffered extracellular solution (pbECS) after 5 minutes causes a rapid recovery of cytoplasmic Ca^2+^ towards basal levels (89% recovery in Mel Im and 88% in Mel Juso, Fig. 1E,F). To exclude potential effects of CAP on the fluorescence dye itself, we measured an emission spectrum before and after 30 s CAP treatment of fura-2 (3 µM) solved in pbECS and observed no relevant changes (Fig. S2).

### The Ca^2+^ influx after direct and indirect CAP treatment mainly originates from intracellular Ca^2+^ sources

**Figure 2:**
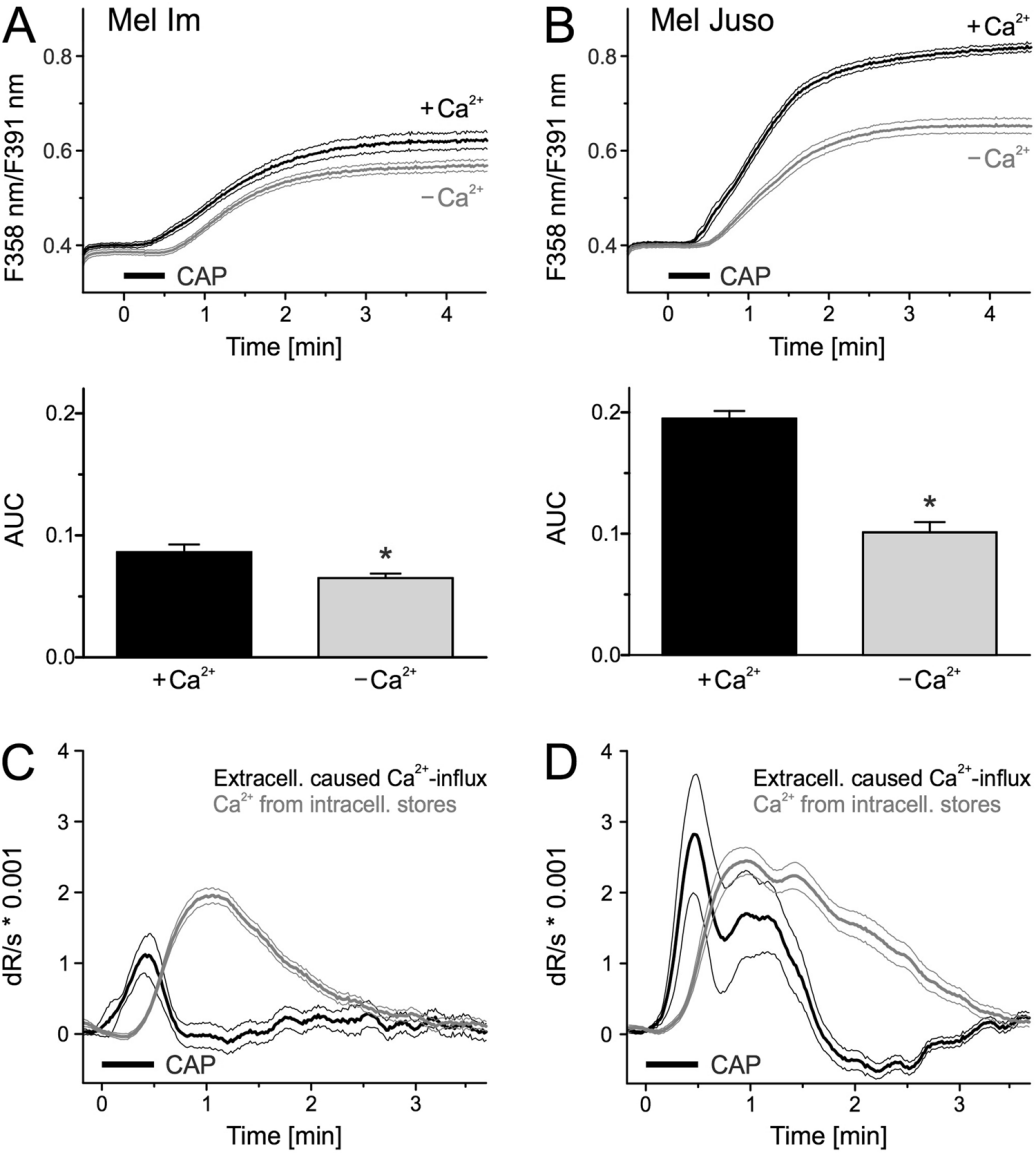
CAP induces Ca2^+^ primarily from intracellular sources. (**A**,**B**) Cytoplasmic Ca^2+^ levels stimulated by 30 s CAP exposure in the presence or absence of extracellular Ca^2+^. Mel Im ((**A**), n = 660 and 464) and Mel Juso. ((**B**), n = 492 and 397) were washed for 5 min in pbECS before CAP in a solution with or without Ca^2+^. (**C**,**D**) Smoothed first derivative (dR/dt) of experiment without extracellular Ca^2+^ (grey trace). The effect due to the presence of extracellular Ca^2+^ is calculated from the difference of experiment with and without extracellular Ca^2+^ (black trace). Data are shown as mean and 99% confidence interval.

Next, we addressed the source of the cytoplasmic Ca^2+^ increase. Without Ca^2+^ in the extracellular environment, both melanoma cell lines showed a reduction in Ca^2+^ increase, but more than half remained (p < 0.001, n = 660 and 464, 25% reduction in Mel Im, p < 0.001, n = 492 and 397, 48% reduction in Mel Juso, t-test independent samples, Fig. 2A,B). This indicates that the majority of the CAP-induced cytoplasmic Ca^2+^ increase stems from intracellular sources. The Ca^2+^ response of cells in the presence of extracellular Ca^2+^ occurred earlier than in experiments without extracellular Ca^2+^. The difference between these two experiments was calculated. The first derivative shows an early and short Ca^2+^ transient depending on extracellular Ca^2+^ sources from the extracellular space. This is in contrast to the later and more pronounced Ca^2+^ transient from intracellular sources, visualized by the first derivative of the experiment without Ca^2+^ (Fig. 2C,D). To quantify this, the peak Ca^2+^ influx determined from extracellular sources occurred earlier than from intracellular sources (both p < 0.001, n = 303 and 320 Mel Juso, n = 260 and 440 Mel Im, t-test independent samples, Fig. 2C,D).

**Figure 3:**
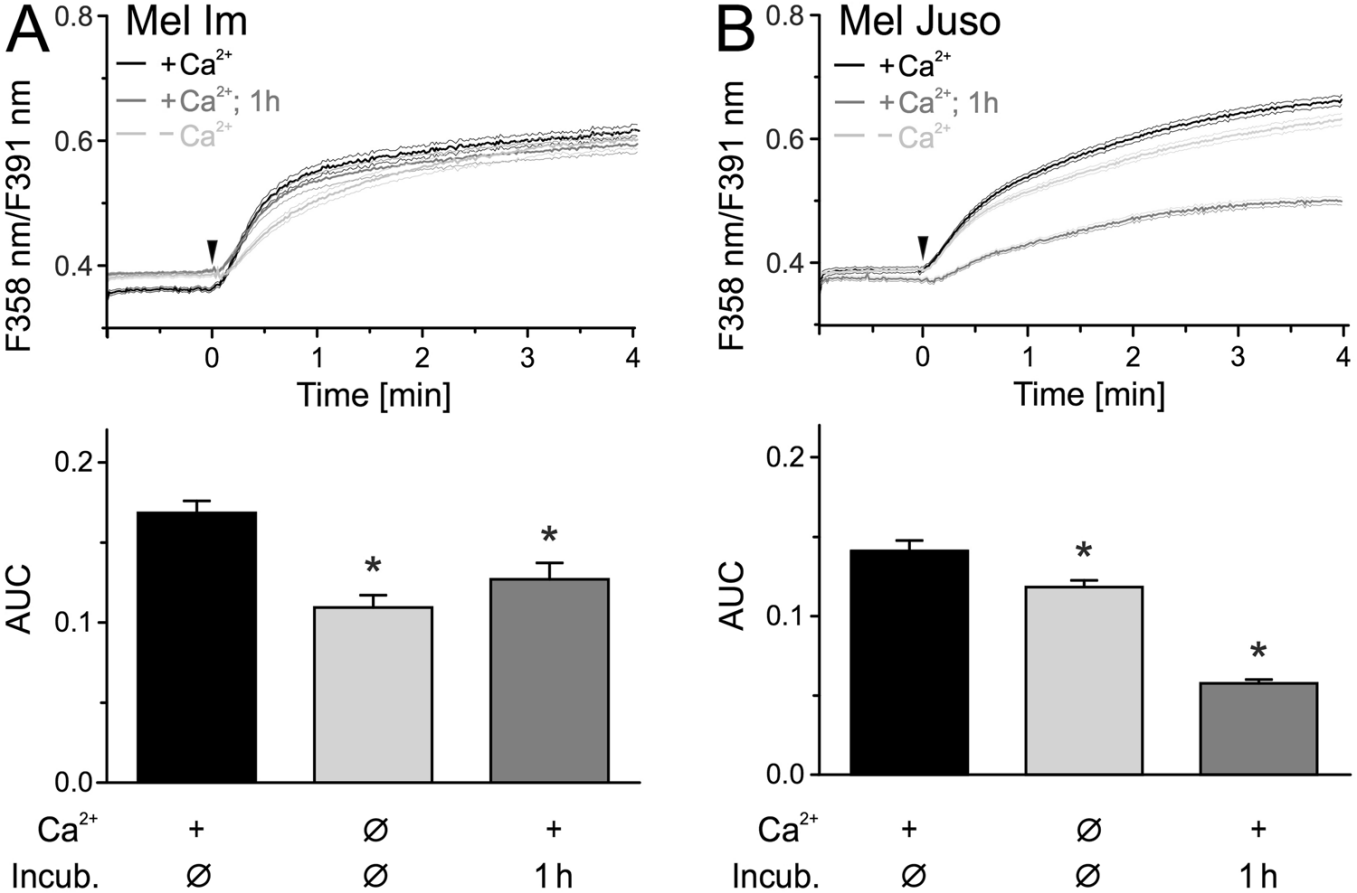
Addition of CAP-pretreated solution onto cells leads to an immediate Ca^2+^ influx. Measurement of cytoplasmic Ca^2+^ using fura-2 AM. CAP exposed pbECS (100 µl) with Ca^2+^ was applied (black traces) onto Mel Im ((**A**), n = 485) and Mel Juso ((**B**), n = 321) 1 min after start of recording (arrowhead). The experiment was repeated with an interval of 1 h between CAP-exposure and application of the solutions (dark grey traces) and in the absence of extracellular Ca^2+^ (light grey trace) ((**A**), n = 274–411; (**B**), n = 366–579). Data are shown as mean and 99% confidence interval.

Besides direct CAP treatment, numerous studies demonstrated that plasma-exposed medium has also antitumor properties^30–32^. We therefore investigated, whether application of a CAP-treated solution also triggers an influx of Ca^2+^ in melanoma cells. PbECS solution applied directly after treatment with CAP for 2 min caused an immediate cytoplasmic Ca^2+^ increase without the delay observed with direct CAP treatment (Fig. 3A,B). Comparing the ratio increase within 30 s and 180 s of CAP, indirect treatment induced a stronger and faster Ca^2+^ response compared to the direct application (Fig. S3). As before, the majority of this response (Mel Im 65%, Mel Juso 84%) was still present in the absence of extracellular Ca^2+^. Interestingly, after incubation for one hour a substantial fraction of the Ca^2+^ increase by the CAP-treated pbECS remained (Mel Im 75%, Mel Juso 41%, Fig. 3A,B). It should be noted that addition of pbECS untreated or pretreated shorter with CAP (30 s and 60 s) had little to no impact on intracellular Ca^2+^ level (Fig. S4). Only 6% of Mel Im reacted to pbECS treated with 60 s CAP and the Ca^2+^ level in the responding cells was back at the basal level within one minute.

We next excluded CAP-induced cell membrane damage as a potential explanation for Ca^2+^ influx from the extracellular space. Propidium iodide (PI), a fluorescent DNA intercalating agent that cannot cross the cell membrane of intact cells, was added to cells directly after CAP treatment. Flow cytometric analysis revealed a similar amount of PI-positive cells in CAP- treated and untreated cells (Fig. S5), which argues against CAP-induced cell membrane damage.

### The endoplasmatic reticulum and the mitochondria are involved in the Ca^2+^ influx

**Figure 4:**
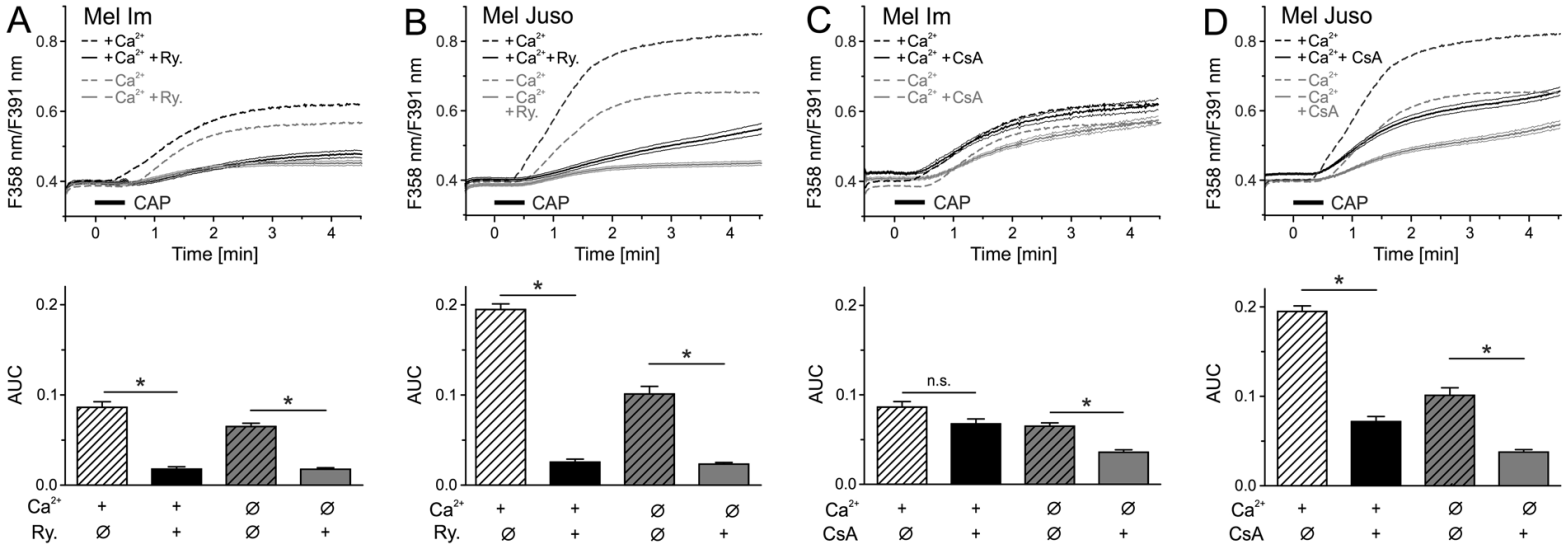
The endoplasmic reticulum and the mitochondria are involved in CAP-induced Ca^2+^ response. Cytoplasmic Ca^2+^ responses induced by 30 s CAP exposure in Mel Im and Mel Juso were reduced by ryanodine (Ry., 30 µM; ((**A**,**B**), n = 324–587) or cyclosporin A (CsA, 0.5 µM; ((**C**,**D**), n = 349–516). Experiments were performed in presence or absence of extracellular Ca^2+^. Data are shown as mean and 99% confidence interval.

Next, the intracellular source of the Ca^2+^ increase was investigated. The endoplasmic reticulum is a main intracellular Ca^2+^ store. When ryanodine receptors were inhibited by ryanodine (Ry, 1 µM) the Ca^2+^ increase by 30 s CAP was reduced to a minimum, both in the presence and absence of extracellular Ca^2+^ (+Ca^2+^ 21%, −Ca^2+^ 27% in Mel Im ANOVA F_(1,2121)_ = 36.6, p < 0.001 each, HSD post-hoc tests, Fig. 4A, +Ca^2+^ 13%, −Ca^2+^ 23% in Mel Juso, ANOVA F_(1,1621)_ = 431, p < 0.001 each, HSD post-hoc tests, Fig. 4B). Further, we investigated a role of the mitochondrial permeability transition pore (mPTP), which can be activated secondary to Ca^2+^ elevation. Inhibiting the mPTP by cyclosporin A (CsA, 0.5 µM) reduced CAP-induced cytosolic Ca^2+^ in Mel Juso cells to 37% (+Ca^2+^ and −Ca^2+^, ANOVA F_(1,1568)_ = 147, p < 0.001 each, HSD post-hoc tests, Fig. 4D). In Mel Im cells, CsA elevated the basal Ca^2+^ level, which restricts conclusions regarding CAP treatment (Fig. 4C).

### The CAP-induced senescence is induced by Ca^2+^ influx

**Figure 5:**
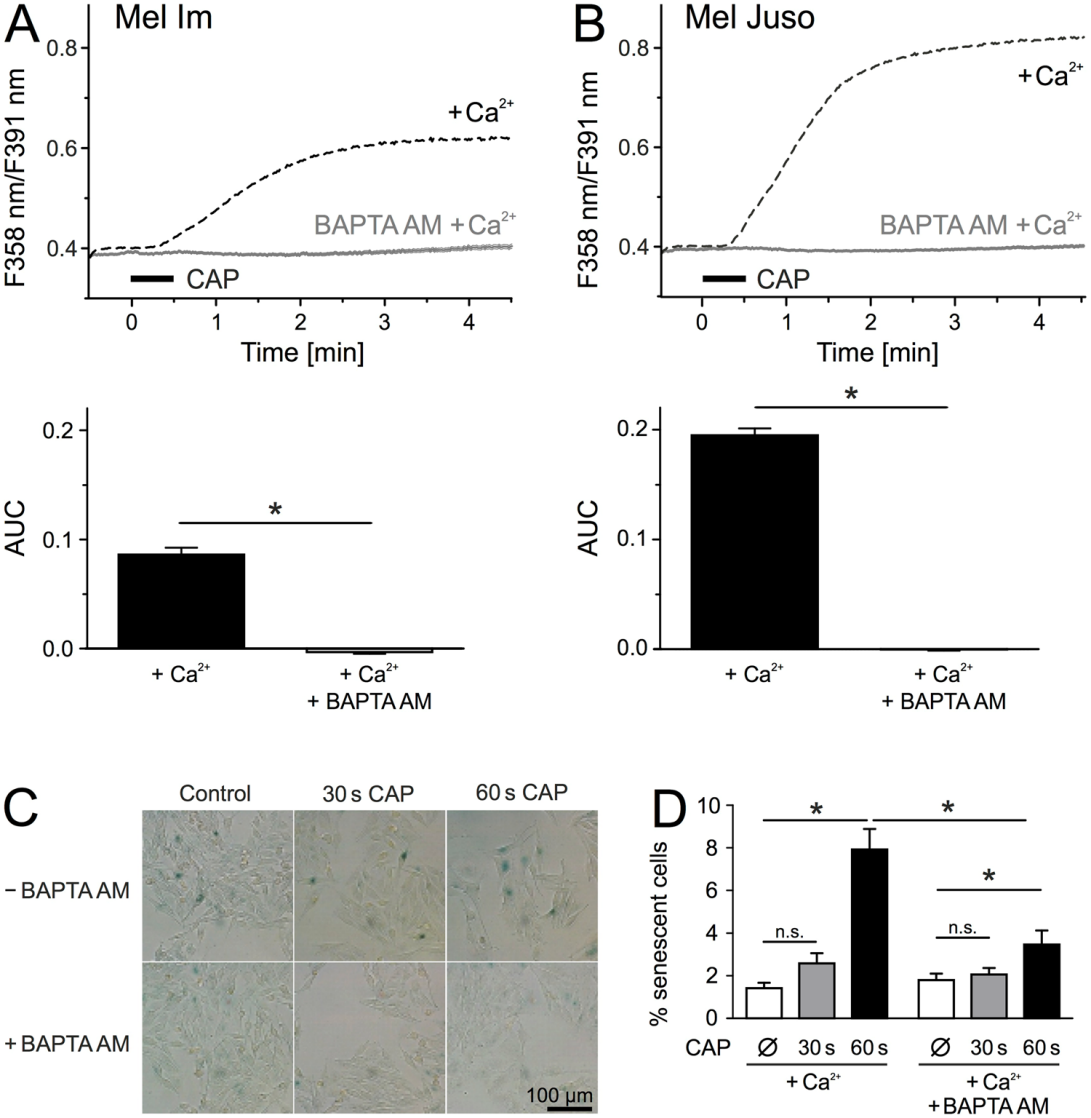
CAP-induced senescence and Ca^2+^ influx are linked to each other. (**A,B**) Mel Im (**A**, n = 389) and Mel Juso (**B**, n = 660) were simultaneously loaded with the Ca^2+^ chelator BAPTA AM (10 µM) and fura-2 AM (3 µM) and cytoplasmic Ca^2+^ was measured during and after 30 s CAP exposure. (**C**,**D**) Senescence-associated ß-galactosidase staining of Mel Juso (n = 20, each from four independent runs) 48 h after CAP exposure for 30 s or 60 s and with or without BAPTA AM (10 µM) pretreatment. (**C**) Representative pictures were taken with a 20 × magnification in bright field illumination. Senescent cells showed a blue staining due to senescence-associated ß-galactosidase activity. Traces are mean and 99% confidence interval (**A**,**B**) and bars are mean ± SEM (**D**).

In a previous study, we observed an induction of senescence in melanoma cells after 1 min CAP exposure^18^. Latest discoveries revealed a correlation between an increase in intracellular Ca^2+^ level and senescence^33,34^. We therefore investigated whether the observed CAP-induced Ca^2+^ influx could be responsible for the senescence induction after CAP exposure. Simultaneously loading with the selective Ca^2+^ chelator BAPTA AM (10 µM) and fura-2 AM (3 µM) abolished the CAP-induced Ca^2+^ influx in both cell lines (p < 0.001 each, Mel Im and Mel Juso, n = 389–660, t-test independent samples, Fig. 5A,B). It should be noted that in the presence of 1 mM Mg^2+^, as is the case with pbECS, the dissociation constant (K_d_) for fura-2 is about 224 nM, whereas BAPTA possesses an K_d_ of 700 nM^35,36^. Thus, a cytosolic Ca^2+^ increase would have been reported by fura-2 also in the presence of BAPTA. By using the senescence-associated ß-galactosidase staining, we next investigated the effect of intracellular Ca^2+^ buffering on CAP-induced senescence in Mel Juso (Fig. 5) and Mel Im (Fig. S6) cells. Unfortunately, analysis after 1 min CAP treatment was not feasible because the cells detached from the 6 well plate after the incubation time of 48 h. In Mel Juso cells, CAP exposure for 1 min raises the amount of senescent cells about 6-fold compared to the untreated control, while 30 s CAP displayed an about 2-fold increase (Fig. 5D). Especially after 1 min CAP, we observed an increased cell size typical for senescent cells, as well as a reduced cell density (Fig. 5C). Treatment with BAPTA AM prior to CAP exposure reduced the amount of senescent cells at both doses, however to a greater extent by 1 min CAP (p < 0.001, n = 20 each from four independent runs, Mann Whitney U-test, Fig. 5D). BAPTA AM pretreatment of Mel Im and Mel Juso further reduced the CAP-induced upregulation of the cell cycle regulators p16 and p21 on mRNA level (Fig. S6A). Interestingly, the mechanisms of senescence induction, however, seems to differ between Mel Im and Mel Juso cells. While p21 mRNA expression was induced after CAP treatment in both cell lines, only Mel Juso cells displayed an increased p16 mRNA level.

## Discussion

In a previous study we demonstrated the potential of CAP produced by a SMD plasma device as a promising new anti-cancer therapy^18^. We observed dose-dependent effects on malignant melanoma cells with 2 min CAP treatment causing apoptosis and 1 min CAP causing senescence.

Here, we revealed Ca^2+^ influx from intracellular sources after direct and indirect CAP treatment in melanoma cells. Ca^2+^ is a key regulator of various signaling pathways, prolonged Ca^2+^ changes affect cell survival and might contribute to the anti-cancer effect of CAP^23,27,37,38^. For instance, Ca^2+^ is a known activator of Ras, a GTP-binding protein, which is mutated in 27% of all human cancers^39,40^. RAS activates the MAPK (mitogen-activated protein kinases) pathway mediating p53-dependet apoptosis^40,41^.

A benefit of the direct CAP treatment was the possibility of investigating even short-lived reactive species which may affect Ca^2+^ homeostasis. Effects of direct CAP treatment on Ca^2+^ influx were not described previously and cytoplasmic Ca^2+^ elevation was so far observed in normal cells, but not in cancer cells after application of CAP-treated solutions^42,43^.

We noted that direct CAP treatment caused an intracellular Ca^2+^ elevation with a few seconds delay indicating that components of the active agent need a few seconds to develop. A previous study described an increase of intracellular Ca^2+^ in mouse fibroblasts with a delay of 70 s, however after indirect CAP treatment^42^. Using indirect CAP treatment, we observed immediate induction of cellular effects supporting our hypotheses that the components develop after CAP in the solution. Differences in the incubation time (60 s vs. 30 s), the cell lines (melanoma cells vs. fibroblasts) as well as in the plasma source (SMD vs. DBD) could explain the partially divergent observations.

Hydrogen peroxide (H_2_O_2_), nitrites (NO_2_^−^) and nitrates (NO_3_) were the most frequent detected species after CAP treatment of different liquids, probably because they are relative stable^44^. Species with lifespans of several minutes were reported to cause the Ca^2+^ influx with extracellular origin^42^. It is likely that these species were just the end-products of several reactions of short-lived species. In addition, the anti-cancer effects of CAP were ascribed to ROS and RNS produced by plasma^20,21^. We observed that CAP-treated solutions induce Ca^2+^ influx even after 1 h, indicating that the components of CAP causing the observed effects are unexpectedly stable. Such species could be the key modulators of the indirect CAP treatment. Three direct CAP exposures (10 s each) caused an additive effect, however a sustained treatment with 30 s CAP led to a stronger increase of intracellular Ca^2+^. This indicates that chemical species with short and long half-life, which need to be determined, contribute to the intracellular Ca^2+^ elevation after direct CAP exposure.

In contrast to studies showing ROS- and RNS-induced Ca^2+^ influx from extracellular sources^42^ we observed CAP-induced Ca^2+^ influx mainly from intracellular Ca^2+^ stores, the endoplasmic reticulum and the mitochondria. Blocking the ryanodine receptor, an important Ca^2+^ release channel of the endoplasmic reticulum, reduced the Ca^2+^ influx after CAP significantly. Inhibition of the mitochondrial permeability transition pore (mPTP) also led to a decrease in CAP-induced Ca^2+^ response. Therefore, we hypothesize that ROS and RNS produced by CAP affect these structures. ROS and RNS are known to modulate Ca^2+^ channels especially through modification of sulfhydryl groups of cysteine residues. For instance, nitric oxide and H_2_O_2_ can activate transient receptor potential (TRP) cation channels like TRPA1 and TRPC5^45,46^. ROS and RNS were further shown to regulate Ca^2+^ release from the endoplasmic reticulum through ryanodine receptors and inositol 1,4,5-trisphosphate receptors^47,48^. Mitochondrial Ca^2+^ overload, oxidative stress and mitochondrial depolarization are important trigger of mPTP opening^49^. The structure of mPTP remains elusive, but several studies described the formation of a mPTP through interaction of mitochondrial cyclophilin D and the adenine nucleotide translocase^49–52^. A further study displayed that oxidative stress induces an intramolecular disulfide bridge in the adenine nucleotide translocase, which in turn enhances the sensitivity of mPTP to Ca^2+^^53^. Hence, it is likely that the observed cytoplasmic Ca^2+^ elevation is caused by CAP-produced ROS and RNS that regulate relevant modulators of Ca^2+^ homeostasis like the ryanodine receptor and the mPTP.

Intracellular Ca^2+^ chelation using a cellular trapped BAPTA led to a complete inhibition of the CAP-induced Ca^2+^ response and abolished the induction of senescence by CAP. We conclude that CAP-induced senescence requires intracellular Ca^2+^ elevation as main inducer. The involvement of cytoplasmic Ca^2+^ elevation on induction of senescence was described in previous studies^33,34^. Furthermore, senescent fibroblasts (passage>25) exhibit an increased intracellular Ca^2+^ level compared to presenescent fibroblasts (passage<15)^54^.

Similar to our observations, buffering of intracellular Ca^2+^ with BAPTA inhibited oxidative stress-induced senescence in human stem cells^33^. As the molecular mechanism the authors describe that a sublethal dose of H_2_O_2_ leads to phospholipase C-dependent production of the second messenger inositol 1,4,5-triphosphate (IP3), which triggers Ca^2+^ release from the endoplasmic reticulum through binding of the 1,4,5-triphosphate receptor (IP3R). In accordance, investigations with human mammary epithelial cells displayed an involvement of IP3R in oncogene-induced and replicative senescence^34^. This group further observed a mitochondrial calcium accumulation, which leads to a decrease of the mitochondrial potential followed by an increase of ROS production. Mitochondrial DNA damage and a decline of ATP production are known consequences of mitochondrial dysfunction and assumed to be involved in senescence induction^33,55,56^. Bentle and colleagues described the involvement of Ca^2+^ in PARP-1 (poly(ADP-ribose) polymerase-1) activation after ROS-induced DNA damage^57^. In addition, intracellular Ca^2+^ chelation with BAPTA diminished DNA-damage in H_2_O_2_-treated human stem cells and thus may attenuate senescence induction^33^.

Our study provides new insights about the molecular cause of CAP-induced senescence in melanoma cells. In summary, we could show that direct and indirect CAP treatment of malignant melanoma cells causes a cytoplasmic Ca^2+^ increase derived from intracellular stores, involving the ryanodine receptor and the mPTP. Induction of senescence, a recently observed effect of CAP treatment of melanoma cells, depends on this Ca^2+^ influx. The involved chemical species still need to be identified, however, our observations already contribute to a better understanding of CAP action on tumor cells. Regarding medical application of CAP, gaining a deeper insight into the molecular background of CAP impact is highly necessary.

## Methods

### Cell culture

The Mel Juso (DSMZ: ACC74) cell line was established from a primary cutaneous melanoma and the Mel Im cell line was derived from a metastasis of malignant melanoma. The cell cultivation has been described previously^58^.

### Plasma device

In this study we use the miniFlatPlaSter plasma device^8^, which was developed for the treatment of tumor cells and tissue by the Max Planck Institute for Extraterrestrial Physics. Based on the SMD technology, the plasma is generated with the ambient air^7^. Device specific technical details can be found in the following publication^18^.

### Chemicals and solutions

The phosphate buffered extracellular solution (pbECS) used in Ca^2+^ imaging experiments consists of following components, listed in mM: 133.0 NaCl, 3.53 KCl, 10 glucose, 1.47 KH_2_PO_4_, 8.06 Na_2_HPO_4_, 1.25 CaCl_2_ × 2H_2_O, 1 MgCl_2_ × 6H_2_0. To obtain a Ca^2+^ free pbECS solution, CaCl_2_ × 2H_2_O was exchanged with 5 mM EGTA. The composition of the extracellular solution (ECS) was previously described^59^. Phosphate buffered saline (PBS) was obtained from Sigma. Further, the following chemicals were used: fura-2 AM (3 µM, Biotium), pluronic F-127 (0.02%, Biotium), ionomycin (2 µM, Enzo Life Sciences), propidium iodide (PI, 10 µg/ml, PromoKine), cyclosporin A (0.5 µM, Merck), ryanodine (30 µM, Santa Cruz), BAPTA AM (10 µM, Merck), etoposide (100 µM, Sigma-Aldrich), DMSO (Sigma-Aldrich). Except PI, all stock solutions were prepared in DMSO and diluted at least 1000-fold for experimental use.

### Calcium imaging

About 200,000 cells were seeded into 35 mm diameter plastic tissue culture dishes (Sarstedt). On the next day the cells were loaded with fura-2 AM (3 µM) in pbECS with 0.02% pluronic for 30 min in an incubator at 37 °C and 8% CO_2_. Next, the cells were washed for 5 min at room temperature in pbECS with or without Ca^2+^ as mentioned. Preexposure to substances including the 5 min wash period are reported in the respective protocols. For direct CAP treatment (Fig. S1A), the solution was removed before recording (33 ± 1 µm fluid layer height of residual fluid, n = 7). A polyethylene plastic foam ring was fixed around the dish forming a closed system. The dish was mounted on an inverse microscope and the applicator of the gravity-driven perfusion system was positioned through a tunnel in the polyethylene foam towards the cells at a distance of ~300 µm. The plasma device was positioned to touch the polyethylene foam. After 30 s of baseline Ca^2+^ measurement, the cells were irradiated with CAP.

For testing CAP-exposed solutions (indirect treatment, Fig. S1B), we irradiated 6 separate 20 µl drops of pbECS with CAP in a tissue culture dish without cells. We used this drop method to achieve a liquid surface area corresponding to direct CAP treatment. During CAP exposure of the test solution, the extracellular solution was removed from the cultured cells and an aluminum ring of 6.5 mm diameter was placed onto the TC dish. After about 30 s of Ca^2+^ measurement, 100 µl of the CAP-exposed pbECS was added to the cells in the ring.

The cells were alternatingly excited at 358 nm and 391 nm and the respective fura-2 fluorescence was recorded. Regions of interest were manually placed inside cells, after background subtraction the F358 nm/F391 nm ratio time course was calculated and averaged for visualization. The area under the curve (AUC) of the period 30–120 s after start of recording was calculated for each cell, the 10 s before application served as reference. Data evaluation as well as the imaging equipment used have been described in further detail previously^59^.

### Senescence-associated ß-galactosidase staining

For detection of senescence, about 110,000 Mel Juso cells were seeded into 35 mm diameter wells (Corning). On the next day, cells were either pretreated or not with BAPTA AM (10 µM) for 30 min in an incubator at 37 °C and 8% CO_2_. After a washing step with PBS, cells were exposed to 30 s CAP and 1 min CAP or remained untreated. As a positive control, cells were incubated with etoposide (100 µM) or with DMSO as a negative control in the same quantity as etoposide (data not shown). After an incubation time of 48 h, cells were fixed and stained according to the Senescence ß-Galactosidase Kit (Cell Signaling). By using an IX83 inverted microscope (Olympus), 5 pictures per well and treatment were captured under bright field illumination with a 20x zoom.

### Detection of membrane damage

About 200,000 cells were seeded into 35 mm diameter wells (Corning) and treated with CAP for 30 s the following day, control cells remained untreated. Propidium iodide (PI) solved in PBS (10 µg/ml) was added directly after CAP exposure. The solution was removed after 5 minutes and the cells were detached with trypsin and collected in FACS tubes. After a washing step with PBS, cells were resuspended in PBS and intracellular PI amount was determined by flow cytometry (FACSCalibur, BD Bioscience).

### RNA isolation and reverse transcription

Total cellular RNA was isolated with E.Z.N.A. ® Total RNA Kit (Omega) according to manufacturer’s instructions 48 h after CAP treatment. cDNAs were generated by reverse transcriptase reaction as described elsewhere^60^.

### Analysis of mRNA expression by real-time PCR

Real-time PCR for p21 and p16 was performed using the LightCycler® 480 II technology (Roche), using following forward and reverse primers from Sigma-Aldrich (p16: fw 5′-GGAGCAGCATGGAGCCTTCGGC-3′; rev 5′-CCACCAGCGTGTCCA GGAAGC-3′; p21: fw 5′-CGA GGC ACC GAG GCA CTC AGA GG-3; rev 5′-CCT GCC TCC TCC CAA CTC ATC CC-3′).

### Statistical analysis

Two groups with at least ten samples were compared with a paired or unpaired t-test. Smaller independent samples were compared by the Mann Whitney test. Repeated measurements and multiple groups were compared by ANOVA and HSD post-hoc tests. Analysis was performed using Statistica 8 (Statsoft, Tulsa, OK, USA) or GraphPad Prism 5 (GraphPad Software Inc., CA, USA). Traces are presented with 99% confidence interval of the mean, other results with mean ± SEM; p < 0.05 was considered significant.

### Data availability

The authors will make materials, data and associated protocols available to readers by mailing to the corresponding author.

## Electronic supplementary material

Supplementary information
