# Supplementary material for: Cold atmospheric plasma causes a calcium influx in melanoma cells triggering CAP-induced senescence
Source: Sci Rep. 2018 Jul 3;8:10048. doi: 10.1038/s41598-018-28443-5 (PMC6030087; doi:10.1038/s41598-018-28443-5)
Supplement: Supplementary file 1 — Supplementary information [file 41598_2018_28443_MOESM1_ESM.pdf]

Cold atmospheric plasma causes a calcium influx in melanoma cells triggering CAP-induced senescence

Christin Schneider[1,#], Lisa Gebhardt[2,#], Stephanie Arndt[3], Sigrid Karrer[3], Julia L. Zimmermann[4], Michael J.M. Fischer[2,5], Anja-Katrin Bosserhoff[1,6*]

Supplementary information

Supplement 1. Schematic figure. Experimental set up for direct **(A)** and indirect **(B)** plasma treatment during Ca^{2+} time lapse imaging. PE stands for polyethylene.

Supplement 2. Excitation spectrum of fura-2 in pbECS is not changed by CAP exposure. The excitation spectrum of fura-2 diluted in pbECS with and without Ca^{2+} was measured before and after treatment with 30 s CAP. The spectra were measured three times and normalized to the isosbestic point at 360 nm. Fluorescence emission was collected above 440 nm (long-pass filter).

Supplement 3. The majority of the Ca^{2+} response is reached faster by indirect CAP treatment than by direct CAP treatment. A,B) Overlay of cytoplasmic Ca^{2+} measurement by direct CAP treatment with 30 s exposure and by indirect CAP treatment with 120 s exposure of Mel Im (**A**, n = 660 and 485) and Mel Juso (**B**, n = 492 and 321). For better illustration, the moment of application of the CAP-treated pbECS solution and the start of direct CAP treatment were set at zero. **C,D)** Calculation of the ratio between Δ 30 s/ Δ 180 s. Data are shown as mean and 99% confidence interval.

Supplement 4. Application of pbECS treated with CAP for 0–120 s. The application of untreated pbECS does not trigger a Ca^{2+} influx in Mel Im (**A,** n = 182) and Mel Juso (**B**, n = 377). In contrast to responses in all cells at the application of pbECS pretreated with CAP for 120 s, only 6% of Mel Im cells (n = 185) react to pbECS pretreated with CAP for 60 s and 2% to CAP for 30 s (n = 252). Mel Juso cells did not respond with Ca^{2+} influx when exposed to pbECS pretreated for 60 s and 30 s (n = 305 and 371).

Supplement 5. CAP exposure causes no membrane damage. Measurement of intracellular propidium iodide positive cells (PI+) by flow cytometry. PI was added direct after 30 s CAP treatment of Mel Im (**A,** n = 3 experiments) and Mel Juso (**B,** n = 3 experiments). The percentage of PI positive cells of untreated cells was compared to the CAP-treated cells. Representative cytograms of PI fluorescence vs. FL1-H (height) channel fluorescence were exemplary shown. Data are shown as mean ± SEM.

Supplement 6. CAP induces senescence in melanoma cells on different ways. A) Mel Im (n = 3 experiments) and Mel Juso (n = 3 experiments) were simultaneously loaded with the Ca^{2+} chelator BAPTA AM (10 µM) and mRNA expression of p21 and p16 was determined 48 h after CAP treatment and compared with the respective controls with or without BAPTA AM pretreatment. **B,C**) Senescence-associated ß-galactosidase staining of Mel Im (n = 5, one run) 48 h after CAP exposure for 30 s with or without BAPTA AM (10 µM) pretreatment. Representative pictures were taken with a 20x magnification in bright field illumination. Senescent cells showed a blue staining due to senescence-associated ß-galactosidase activity. Data are mean ± SEM (**C**).

S1

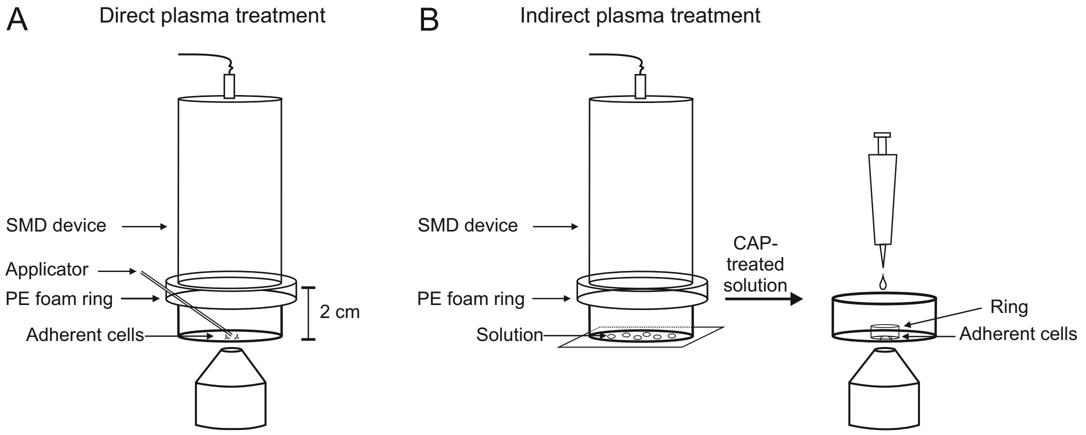
A
Direct plasma treatment
SMD device
Applicator
PE foam ring
Adherent cells
2 cm
B
Indirect plasma treatment
SMD device
PE foam ring
Solution
CAP-
treated
solution
Ring
Adherent cells

S2

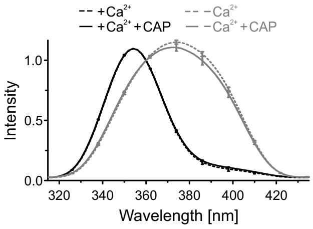

+Ca2+
−Ca2+
+Ca2+ +CAP
−Ca2+ +CAP
Intensity
1.0
0.5
0.0
320
340
360
380
400
420
Wavelength [nm]

S3

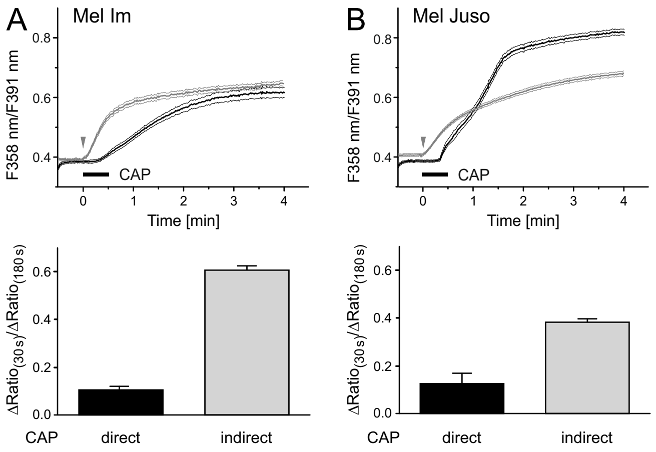

A
Mel Im
B
Mel Juso
F358 nm/F391 nm
0.4
0.6
0.8
CAP
0
1
2
3
4
Time [min]
ΔRatio(30 s)/ΔRatio(180 s)
0.0
0.2
0.4
0.6
CAP
direct
indirect

S4

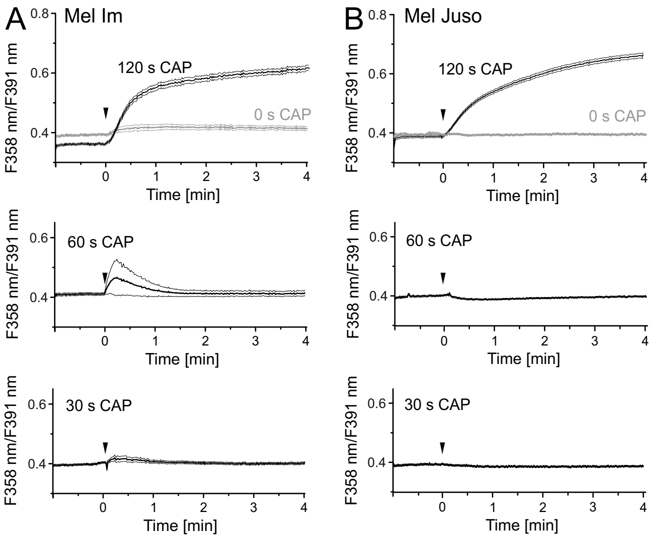

A
Mel Im
120 s CAP
0 s CAP
60 s CAP
30 s CAP
B
Mel Juso
F358 nm/F391 nm
Time [min]

S5

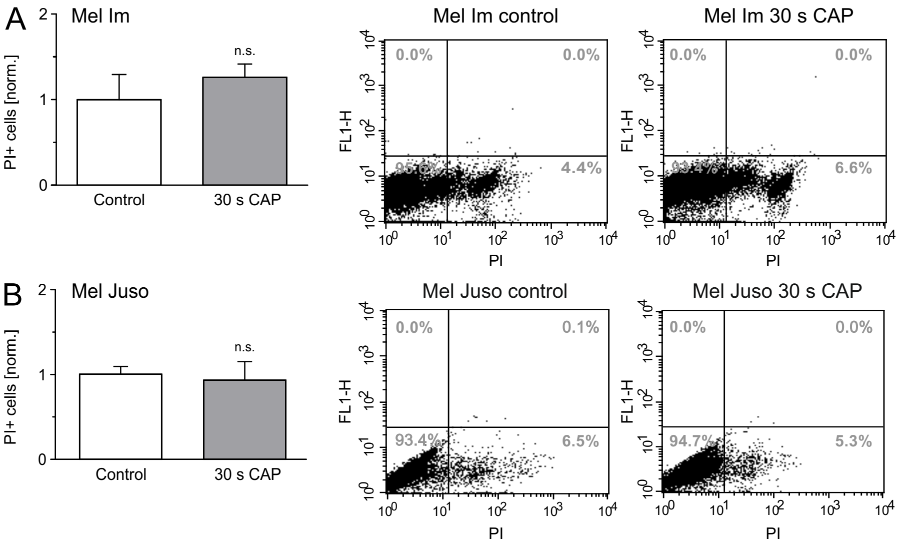

A
Mel Im
PI+ cells [norm.]
2
1
0
n.s.
Control
30 s CAP
Mel Im control
Mel Im 30 s CAP
FL1-H
PI
0.0%
0.0%
4.4%
0.0%
0.0%
6.6%
B
Mel Juso
n.s.
Control
30 s CAP
Mel Juso control
Mel Juso 30 s CAP
0.0%
0.1%
93.4%
6.5%
0.0%
0.0%
94.7%
5.3%

S6

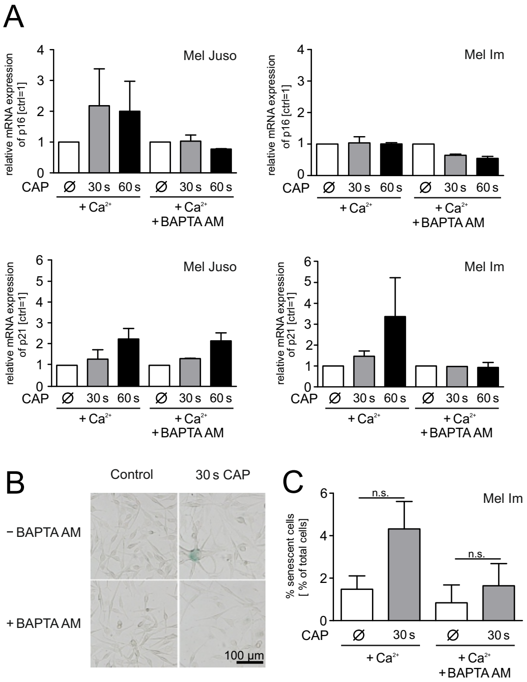
A
Mel Juso
Mel Im
relative mRNA expression of p16 [ctrl=1]
relative mRNA expression of p21 [ctrl=1]
CAP
Ø
30 s
60 s
+ Ca2+
+ Ca2+ + BAPTA AM
B
Control
30 s CAP
− BAPTA AM
+ BAPTA AM
100 µm
C
Mel Im
% senescent cells [% of total cells]
n.s.
